# CT-Based Attenuation Correction in I-123-Ioflupane SPECT

**DOI:** 10.1371/journal.pone.0108328

**Published:** 2014-09-30

**Authors:** Catharina Lange, Anita Seese, Sarah Schwarzenböck, Karen Steinhoff, Bert Umland-Seidler, Bernd J. Krause, Winfried Brenner, Osama Sabri, Jens Kurth, Swen Hesse, Ralph Buchert

**Affiliations:** 1 Department of Nuclear Medicine, Charité – Universitätsmedizin Berlin, Berlin, Germany; 2 Department of Nuclear Medicine, Universitätsklinikum Leipzig, Leipzig, Germany; 3 Department of Nuclear Medicine, Universitätsmedizin Rostock, Rostock, Germany; 4 GE Healthcare Buchler GmbH & Co. KG, Munich, Germany; University of Nebraska Medical Center, United States of America

## Abstract

**Purpose:**

Attenuation correction (AC) based on low-dose computed tomography (CT) could be more accurate in brain single-photon emission computed tomography (SPECT) than the widely used Chang method, and, therefore, has the potential to improve both semi-quantitative analysis and visual image interpretation. The present study evaluated CT-based AC for dopamine transporter SPECT with I-123-ioflupane.

**Materials and methods:**

Sixty-two consecutive patients in whom I-123-ioflupane SPECT including low-dose CT had been performed were recruited retrospectively at 3 centres. For each patient, 3 different SPECT images were reconstructed: without AC, with Chang AC and with CT-based AC. Distribution volume ratio (DVR) images were obtained by scaling voxel intensities using the whole brain without striata as reference. For assessing the impact of AC on semi-quantitative analysis, specific-to-background ratios (SBR) in caudate and putamen were obtained by fully automated SPM8-based region of interest (ROI) analysis and tested for their diagnostic power using receiver-operator-characteristic (ROC) analysis. For assessing the impact of AC on visual image reading, screenshots of stereotactically normalized DVR images presented in randomized order were interpreted independently by two raters at each centre.

**Results:**

CT-based AC resulted in intermediate SBRs about half way between no AC and Chang. Maximum area under the ROC curve was achieved by the putamen SBR, with negligible impact of AC (0.924, 0.935 and 0.938 for no, CT-based and Chang AC). Diagnostic accuracy of visual interpretation also did not depend on AC.

**Conclusions:**

The impact of CT-based versus Chang AC on the interpretation of I-123-ioflupane SPECT is negligible. Therefore, CT-based AC cannot be recommended for routine use in clinical patient care, not least because of the additional radiation exposure.

## Introduction

Single photon emission computed tomography (SPECT) with I-123-labelled cocaine ligands for the presynaptic dopamine transporter (DAT) is widely used for the diagnosis of Parkinsonian syndromes and for differentiation between Alzheimer’s and Lewy body disease [1]–[8]. It is also used for monitoring progression of presynaptic dopaminergic degeneration [9]. The interpretation of DAT SPECT is based on visual evaluation of the images supported by semi-quantitative analysis [10]–[16] both of which are affected by photon attenuation in the head [17].

There are several methods for attenuation correction (AC) in brain SPECT. The most widely used method, proposed by Chang [18], is based on post-processing of images reconstructed without attenuation correction. However, this method has three limitations. First, delineation of the outer contour of the head is required which is prone to errors in case of both manual and automatic threshold-based methods [19]. Second, Chang’s method assumes a uniform attenuation coefficient throughout the whole head which is an approximation only. Third, it is an approximation even if the actual attenuation coefficient is uniform. It is strictly correct only for a point source (at least in its non-iterative form).

Errors associated with these limitations of the Chang AC might be avoided by modelling attenuation within the reconstruction process using the correct attenuation map, for example measured by low-dose x-ray computed tomography (CT). Whereas CT-based AC has become standard in positron emission tomography (PET) of the brain, it is not widely used in brain SPECT, despite the increasing availability of dual-modality SPECT/CT systems. CT-based AC could provide more accurate AC than the Chang method and, therefore, has the potential to improve both semi-quantitative analysis and visual interpretation of DAT SPECT.

The aim of the present study was to compare CT-based AC and Chang AC in dopamine transporter SPECT with N-ω-fluoropropyl-2β-carbomethoxy-3β-(4-I-123-iodophenyl)nortropane (I-123-ioflupane). The impact of the AC method was assessed on both semi-quantitative analysis and visual evaluation of the SPECT images by independent readers blinded for clinical information.

## Materials and Methods

### 1. Phantom studies

SPECT/CT of an anthropomorphic phantom for quantitative SPECT and PET imaging of the striatum (Striatal Phantom, Alderson, Radiology Support Devices Inc., Long Beach, CA, USA) was performed at each of 3 centers (Berlin, Leipzig, Rostock). The striata were filled symmetrically with about 40 kBq*/*ml I-123 solution. The background was filled with about 5 kBq*/*ml. Actual activity concentrations in striatum and background were determined by measuring aliquots in a well counter.

### 2. Subjects

The study included 62 patients who had received SPECT with I-123-ioflupane including low-dose CT in clinical routine patient care for the diagnosis of a Parkinsonian syndrome (Berlin n = 21, Leipzig n = 22 and Rostock n = 19). Consecutive patients were included at each centre without any further inclusion or exclusion criteria.

### 3. Ethics statement

The protocol of this retrospective study had been approved by the Ethics Committee at Rostock University Medical Centre (reference number A 2013-0144) and the Ethics Committee at Charité University Medical Centre Berlin (reference number EA1/326/13). The protocol complied with the Declaration of Helsinki. All patients had given written consent for retrospective analyses of their data.

### 4. SPECT imaging

SPECT imaging including low-dose CT had been performed with a Symbia T6 dual-head SPECT/CT system equipped with low energy high resolution parallel-hole collimators at each centre. The acquisition protocol, based on procedure guidelines for I-123-labelled dopamine transporter ligands and the ENC-DAT study [11], [20], [21], was the same at each centre except for small differences in the energy window at the I-123 peak and the two additional energy windows for scatter correction (SC; energy windows in keV: Berlin [141.66, 173.14], [130.64 141.66], [173.14, 182.58], Leipzig: [147.07, 170.93], [123.22, 147.07], [170.93, 194.77], Rostock: [140.91, 172.91], [129.95, 140.91], [172.23, 181.62]). These differences were taken into account by adjustment of the weight factors during scatter correction (s. below).

Patients did not use medication or drugs known to strongly interact with I-123-ioflupane SPECT [22], [23]. 150 to 200 MBq I-123-ioflupane were injected intravenously as a slow bolus after blocking the thyroid gland by oral administration of perchlorate. SPECT imaging was started between 3 and 4 hours post injection. The rotation radius was adjusted for each patient so that collimator-patient distance was as small as possible. The duration of the SPECT acquisition was 30 minutes (step-and-shoot mode, matrix 128×128, angular range 180°, angular sampling 3°, zoom 1.23). A low-dose CT was performed immediately after the SPECT (130 keV, 50 mAs, 3 mm slice thickness).

The anonymized raw data from all centres were transferred to one centre (Berlin) for centralized image reconstruction and processing. Transversal images were reconstructed using the 3D ordered-subset expectation-maximisation algorithm ‘Flash-3D’ provided by the Siemens scanner software (15 subsets and 6 iterations) [24]–[26]. Flash-3D allows CT-based AC, uniform AC according to Chang and no AC. Spatial resolution in the reconstructed images was about 8 mm full-width-at-half-maximum, voxel size was 3.9×3.9×3.9 mm^3^.

For conversion of Hounsfield units to linear attenuation coefficients (LAC) in CT-based AC, the narrow-beam LAC of µ = 0.148 cm^−1^ was assumed for soft tissue, because SC using the triple energy window approach implemented in the scanner software was applied in all cases [27]. In order to achieve best comparability between Chang and CT-based AC, Chang AC was performed with the same LAC, i.e. µ = 0.148 cm^−1^. For Chang AC, the threshold-based automatic delineation of the head contour implemented in the system software was used. The threshold was optimized in each individual patient so that the best match with the outer contour of the scalp was obtained according to visual inspection.

Image reconstruction without AC, with CT-based AC and Chang AC for each patient resulted in a total of 3 • 62 = 186 SPECT images.

### 5. Data evaluation

The impact of the AC method was assessed on both semi-quantitative analysis and visual evaluation of the SPECT images.

#### 5.1. Impact on semi-quantitative analysis

**Figure 1:**
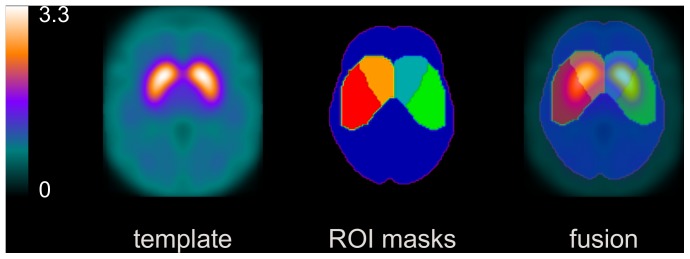
Custom-made I-123-ioflupane template. Transversal slice (**left**). ROIs for left/right caudate and putamen used for hottest voxel analysis, and ROI for the reference region used for intensity scaling, all defined in MNI space (**middle**). The union of caudate and putamen ROI was used as ROI for the whole striatum. Fusion image (**right**).

For semi-quantitative assessment of DAT availability, the I-123-ioflupane SPECT image of each individual patient was stereotactically normalized into the anatomical space of the Montreal Neurological Institute (MNI) using the normalization tool of the freely available Statistical Parametric Mapping software package SPM (version SPM8, Wellcome Trust Centre for Neuroimaging, Institute of Neurology, UCL, London, UK) [28] and a custom-made tracer-specific template (Fig. 1). The stereotactically normalized I-123-ioflupane uptake image was scaled to the 75^th^ percentile of the voxel intensities in the whole brain without striata as reference region (Fig. 1), i.e. the intensity value of each voxel was divided by the 75^th^ percentile of the voxel intensities in the reference region [29]. The voxel intensity of the scaled SPECT image represents the distribution volume ratio (DVR) as semi-quantitative measure for local DAT availability.

The DVRs in caudate and putamen were obtained by ‘hottest voxel analysis’ in large predefined ROIs for left/right caudate and left/right putamen in MNI space (Fig. 1). The number of hottest voxels to be averaged was fixed to a total volume of 5 ml for the caudate and 10 ml for the putamen. The DVR of the whole striatum was obtained by averaging over the 15 ml hottest voxels in the union of the caudate and the putamen ROI. DVRs were converted to specific binding ratios (SBR) according to the formula SBR = DVR –1. The SBR may be considered an estimate of the nondisplaceable binding potential and, therefore, to be proportional to the density of DAT available for binding of I-123-ioflupane [30].

Caudate-to-putamen ratio ( = SBR_caudate_/SBR_putamen_) and left/right asymmetry (asym(%) = 200 • abs[(SBR_left_ − SBR_right_)/(SBR_left_+SBR_right_)]) were considered in addition to SBRs.

#### 5.2. Impact on visual scoring

**Figure 2:**
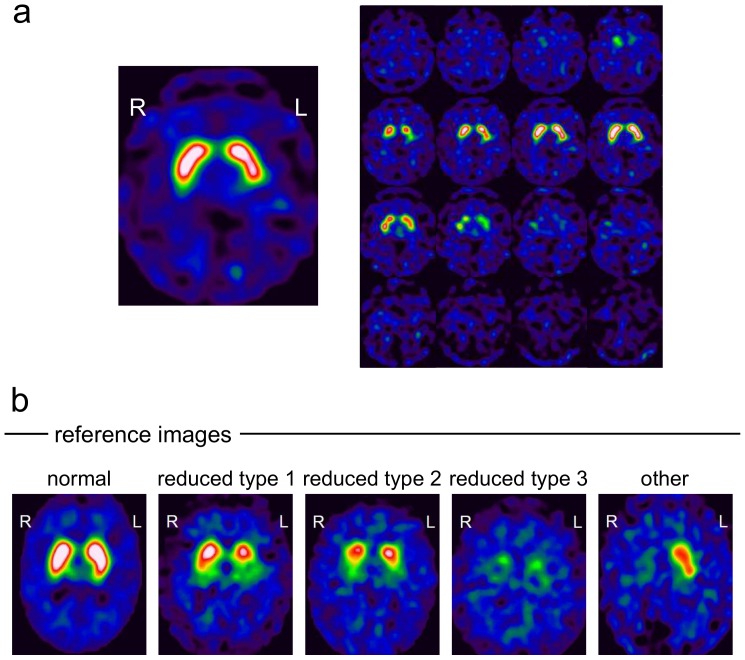
Example page from the pdf document for visual scoring. The pdf document comprised one page for each I-123-ioflupane SPECT image showing a 12 mm thick slab (**a,** **left**) and 4x4 slices of 4 mm thickness (**a,** **right**). Example I-123-ioflupane SPECTs used as reference images for the visual scoring (**b**).

For retrospective visual interpretation of the SPECT images, a pdf document was prepared with 186 pages, one page for each SPECT image (Fig. 2 a). The SPECT images were anonymized and presented in randomized order. To guarantee comparable display conditions, the upper threshold of the colour table was adjusted separately for each AC method. For Chang AC, the upper threshold of the colour table was set to 5.50. For CT-based AC, the upper threshold of the colour table was scaled by the DVR of the caudate (mean over left and right hemisphere) averaged over all patients, i.e. threshold(CT) = avgDVR(CT)/avgDVR(Chang) • threshold(Chang), analogously for no AC. The lower threshold of the colour table was set to one-tenth of the upper threshold in all cases.

Visual scoring of DAT availability was performed on a patient base referring to Benamer et al. [31] using the following 5-score:

‘normal’: clear delineation of the whole striatum on both sides, some minor global reduction of tracer uptake is allowed as well as some minor left/right asymmetry (‘only big effects indicate pathology’)
‘reduced type 1’: distinct reduction in the putamen in one hemisphere (‘big’ effect), the striatum in the other hemisphere still more or less normal
‘reduced type 2’: distinct reduction in both putamina, still some uptake in the caudate nuclei
‘reduced type 3’: essentially no uptake in both striata
‘reduced other’: clear reduction of tracer uptake, but the pattern does not match type 1, 2 or 3. This category was added to the Benamer scheme in order to account for atypical patterns, for example due to vascular pathology

The examples shown in Fig. 2 b were provided to the raters.

The certainty with respect to the differentiation between normal and reduced (including all types of reduction) was scored in addition to DAT availability. A 5-score was used ranging from 1 = ‘very sure’ to 5 = ‘very unsure‘.

Image quality was scored from 1 = ‘very good‘ to 5 = ‘very bad‘ using the anatomical delineation of the striata and the level of statistical noise in the background as criteria.

Visual scoring was performed by two independent raters at each of the 3 centres (image quality in Leipzig only). In addition, the two raters at each centre reached a consensus with respect to DAT availability in those cases in which they had disagreed.

### 6. Statistical methods

Statistics were performed with IBM SPSS Statistics (version 21, IBM Corp., Armonk, NY, USA).

Concerning the results of the semi-quantitative analysis, the effect of the AC method on the SBRs, left/right asymmetries and caudate-to-putamen ratio was first assessed by Bland-Altman plots [32]. Then the impact of AC on the diagnostic power of the semi-quantitative parameters was tested using receiver-operator characteristic (ROC) analysis. The classification of DAT availability in the written report in the patient’s file served as gold standard (22 patients with normal DAT availability, 40 patients with reduced DAT availability). The area under the ROC curve (AUC) was used as performance measure. Multiple binary logistic regression (forward conditional) was performed to analyze the diagnostic power of combinations of SBR, asymmetry and caudate-to-putamen ratio. Only the putamen SBR was included in the multiple regression analysis, since there was a strong correlation between caudate and putamen SBR (Pearson correlation coefficient = 0.87, p = 0.000, Chang AC) so that it was not advisable to include both as independent parameters. The putamen SBR was selected, because it showed higher diagnostic power than the caudate SBR in the univariate ROC analyses.

Concerning the results of the visual reading, inter-rater agreement was quantified by Cohen’s unweighted κ [33], both for the full 5-score for DAT availability and the binary score derived from the 5-score by subsuming ‘reduced type 1, 2, 3′ and ‘reduced other’ into one single category ‘reduced’. Agreement was assessed between the 6 raters and between the 3 consensus scores. Diagnostic accuracy of the visual scoring was characterized by the % agreement with the gold standard.

The effect of the AC method on the certainty in differentiation between normal and reduced DAT availability as well as on image quality was tested by the general linear model for repeated measures. The DAT availability (normal or reduced) according to the report was added to the model as within-subject factor.

Potential covariates such as age or body mass index were not taken into account [34].

## Results

### 1. Impact on semi-quantitative analysis

Results of the semi-quantitative analysis of the phantom and patient studies are summarized in Table 1 and Table 2, respectively.

**Table 1:** Results of the semi-quantitative analysis of the phantom studies.

		actual SBR	measured SBR		
centre	method		left	right	measured/actual SBR (%)^a^
Berlin	no AC	5.47	2.30	2.09	40.1
	CT-based	5.47	2.55	2.37	45.0
	Chang	5.47	2.88	2.62	50.3
Leipzig	no AC	5.74	2.57	2.36	42.9
	CT-based	5.74	2.92	2.53	47.5
	Chang	5.74	3.04	2.88	51.6
Rostock	no AC	5.19	2.36	2.40	45.9
	CT-based	5.19	2.60	2.64	50.5
	Chang	5.19	2.85	2.86	55.0
mean	no AC				43.0±2.9
	CT-based				47.6±2.8
	Chang				52.3±2.4

The specific-to-background ratio (SBR) was measured using the same whole striatum ROIs as in the patient studies (Fig. 1).

a measured SBR: mean over left and right hemisphere.

**Table 2:** Results of the semi-quantitative analysis of the patient studies.

	reduced DAT availability (n = 40)			normal DAT availability (n = 22)		
	no AC	CT-based	Chang	no AC	CT-based	Chang
SBR caudate	1.85±0.75	2.03±0.71	2.42±0.83	3.01±0.51	3.15±0.54	3.67±0.65
SBR putamen	0.80±0.41	0.96±0.44	1.07±0.46	1.64±0.21	1.89±0.26	2.00±0.25
SBR striatum	1.33±0.57	1.51±0.57	1.78±0.65	2.36±0.35	2.54±0.39	2.91±0.45
caudate-to-putamen ratio	2.67±0.84	2.37±0.61	2.53±0.64	1.93±0.30	1.75±0.23	1.91±0.28
asymmetry caudate (%)	15.17±11.95	14.92±12.19	12.59±10.82	7.06±6.14	6.95±5.53	6.52±5.87
Asymmetry putamen (%)	23.28±24.18	20.50±15.81	18.82±16.84	9.11±7.91	7.15±7.81	9.06±8.29
asymmetry striatum (%)	15.92±12.94	15.43±11.58	13.35±10.65	5.65±5.13	6.00±4.55	5.28±5.15

Given are mean values ±1 standard deviation (minimum over both hemispheres for the SBRs, maximum over both hemispheres for the caudate-to-putamen ratio). Subjects were categorized as ‘reduced’ or ‘normal’ DAT availability according to the written report in the patient’s file.

In the phantom studies, the actual SBR was strongly underestimated in all cases, by about 57%, 52% and 48% with no, CT-based and Chang AC (Table 1), i.e. CT-based AC resulted in intermediate SBRs about half way between no AC and Chang AC.

**Figure 3:**
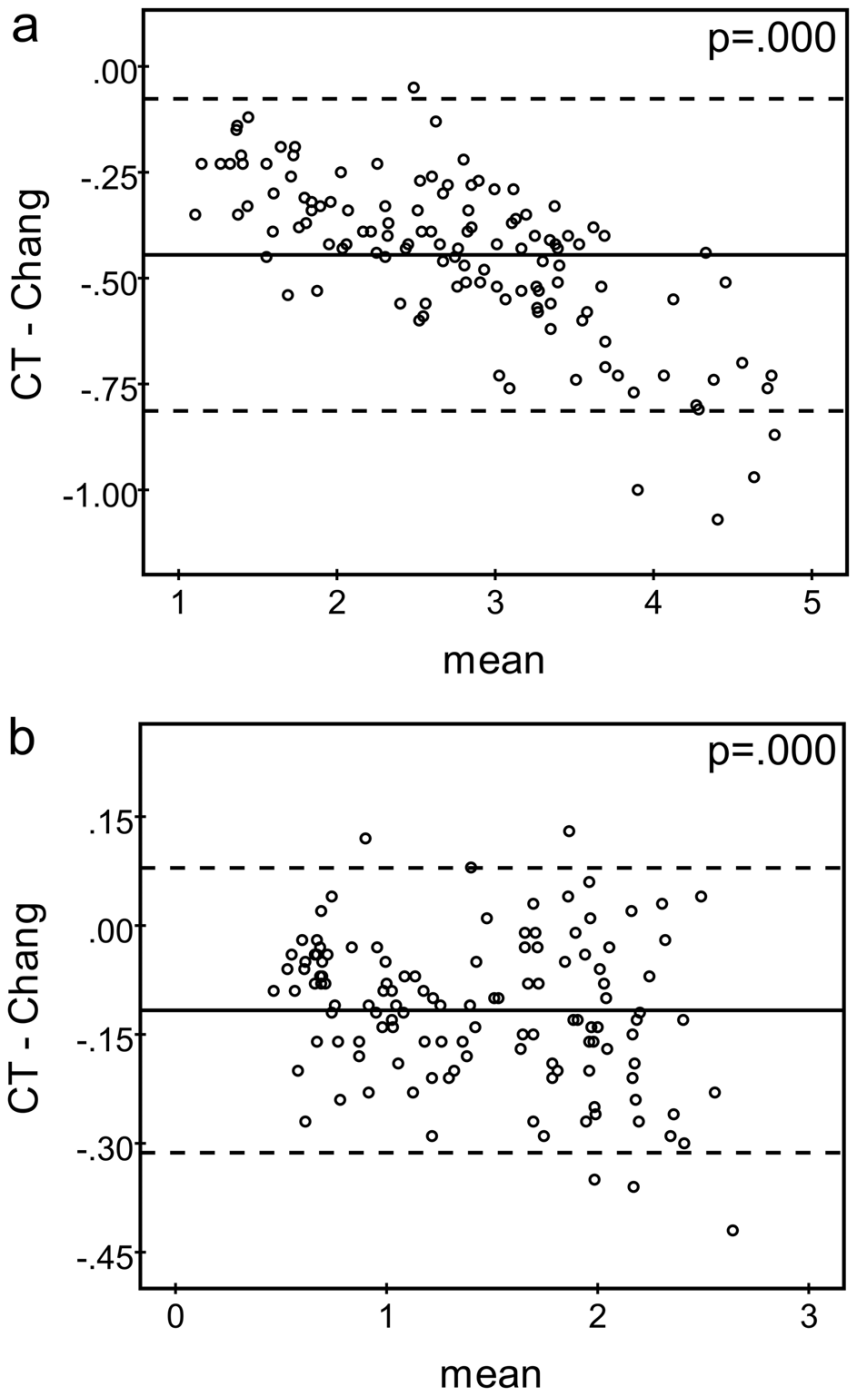
Bland-Altman plots comparing the SBR of the caudate (a) and the putamen (b) between CT-based and Chang AC (SBRs of both hemispheres were included independently, i.e. n = 124). Different scales were chosen for abscissae and ordinates in **a** and **b** for display purposes. The horizontal continuous line represents the mean difference, the dashed lines indicate the 95% confidence interval. The given p-value corresponds to the one-sample t-test for zero mean.

The latter was confirmed in the patient studies (Table 2, Fig. 3). Chang AC resulted in an overestimation of the caudate SBR compared to CT-based AC that was increasing with increasing SBR (Fig. 3 a). The mean difference (CT-based – Chang) was −0.445±0.188 (one sample t-test for zero mean: p<0.0005). The mean difference of the putamen SBR was −0.117±0.100 (p<0.0005). The mean difference of left/right asymmetry in the caudate was 1.66±4.94% (p = 0.011), in the putamen 0.41±7.16% (p = 0.652), and in the whole striatum 1.60±4.79% (p = 0.011). The mean difference of the caudate-to-putamen ratio was −0.15±0.15 (p<0.0005).

Univariate analysis of variance with the SBR (minimum over both hemispheres) as dependent variable and region of interest (caudate, putamen), DAT status (normal or reduced according to the written report in the patient’s file) and centre (Berlin, Leipzig, Rostock) as fixed factors revealed highly significant effects of the region of interest and DAT status (both p<0.0005), whereas there was no centre effect, independent of the AC method.

**Figure 4:**
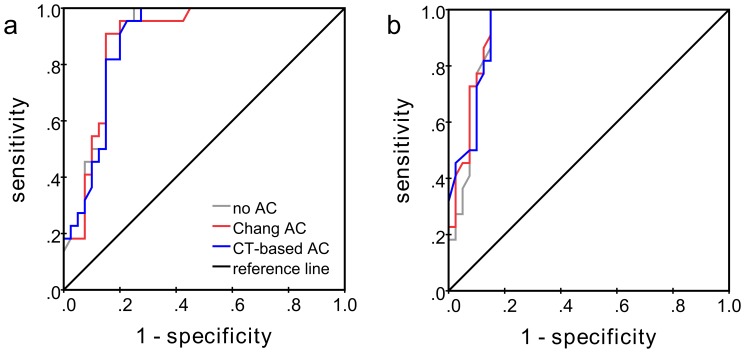
ROC curves for the differentiation between reduced and normal DAT availability by the SBR of the caudate (a) and the putamen (b) (minimum over both hemispheres).

ROC curves for the differentiation between reduced and normal DAT availability by the SBRs are shown in Fig. 4. The putamen provided larger AUC than the caudate. The impact of AC on the AUC was very small: AUC for the caudate 0.892 versus 0.886 versus 0.887, and for the putamen 0.924 versus 0.935 versus 0.938 for no AC, CT-based AC and Chang AC, respectively. Asymmetries and caudate-to-putamen ratio provided less diagnostic power than the SBR (AUC ≤0.875).

The binary regression model included only the putamen SBR (minimum over both hemispheres). The asymmetry of the SBR in the whole striatum and the caudate-to-putamen ratio (maximum over both hemispheres) were not included, independent of the AC method (asymmetry: p≥0.415, caudate-to-putamen ratio: p≥0.179). The number of false classified patients was 8 for no AC and 9 for both CT-based and Chang AC.

### 2. Impact on visual scoring

Inter-rater agreement of the visual scoring of DAT availability was higher (i) for the consensus scores compared to the scores of individual raters and (ii) for the binary score compared to the 5-score for DAT availability (Table 3). The impact of the AC method was very small (Table 3).

**Table 3:** Cohen’s unweighted κ (mean ±1 standard deviation) for inter-rater agreement of visual scoring of DAT availability.

	individual raters			consensus scores		
	(15 pairs of raters)			(3 pairs of consensi)		
	no AC	CT-based	Chang	no AC	CT-based	Chang
5-score	0.71±0.11	0.70±0.10	0.72±0.09	0.80±0.14	0.78±0.04	0.80±0.06
binary score	0.73±0.13	0.77±0.12	0.76±0.11	0.90±0.05	0.82±0.02	0.81±0.06

Percent agreement of the visual score for DAT availability between CT-based and Chang AC was 81.5±7.9% for the full 5-score and 92.7±2.7% for the binary differentiation between normal and reduced DAT availability (averaged over the 6 individual raters). The consensus scores of the two raters at each centre showed very similar agreement of 83.3±6.5% for the 5-score and 91.4±0.9% for the binary decision. Diagnostic accuracy averaged over all raters was 81.7±3.2%, 81.5±4.9% and 79.6±1.7% for no AC, CT-based and Chang AC, respectively. Diagnostic accuracy of the consensus was 83.3±1.9%, 83.9±1.6% and 79.6±2.5%.

The AC method had no significant effect on the raters’ certainty in the differentiation between reduced and normal DAT availability (p = 0.922). However, there was a significant effect of the status of DAT availability: the certainty of the visual scoring was lower in patients with normal DAT availability (score averaged over all raters and all AC methods: 2.40±0.83 versus 1.24±0.60 in patients with normal and reduced DAT availability, p<0.0005).

Image quality appeared slightly reduced without AC (score = 2.36±0.54), but very similar for the two AC methods (2.16±0.44 and 2.22±0.53 for CT-based and Chang AC, p = 0.393).

## Discussion

The present study compared CT-based AC and Chang AC for dopamine transporter SPECT with I-123-ioflupane with respect to both semi-quantitative and visual analysis. The same reconstruction algorithm with the same parameter settings was used with both AC methods in order to simplify the interpretation of observed differences in reconstructed images (as effects of AC), in contrast to most previous studies which used Chang AC with filtered backprojection whereas CT-based AC was implemented within an iterative reconstruction algorithm [24]–[26]. It is well known that the reconstruction algorithm itself, in this case iterative reconstruction versus filtered backprojection, has an impact on the SPECT images. In the present study, the 3D ordered-subset expectation-maximisation algorithm ‘Flash-3D’ was used with the same number of subsets (15) and iterations (6) in all cases. This might have introduced a systematic effect not directly related to AC, since the speed of convergence of iterative reconstruction depends on whether attenuation is modelled or not. However, this effect is expected to be rather small in the present setting and, therefore, was neglected.

Concerning the results of the semi-quantitative analysis in the present study, CT-based AC resulted in intermediate SBRs about half way between no AC and Chang AC. However, the differences were rather small. For example, the increase of the SBR of the whole striatum by CT-based AC compared to no AC was only about 12% in the phantom studies (Table 1). This is explained by the fact that (i) the effect of photon attenuation is considerably smaller in I-123-ioflupane SPECT than in brain PET, due to the shorter path length to be covered by the single photon in SPECT compared to the total path length of both photons in PET which overcompensates the higher attenuation coefficient in SPECT, and that (ii) the effect of attenuation cancels to some extent during the computation of the SBRs as uptake ratios.

**Figure 5:**
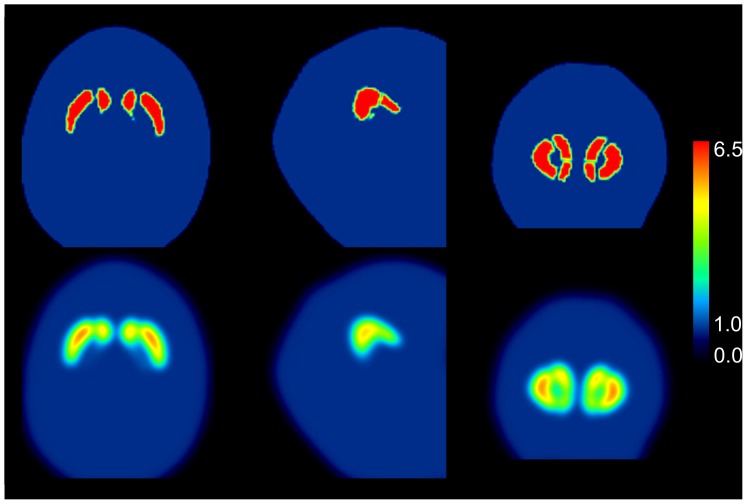
SPECT underestimates the true activity concentration in small structures such as the striatum and its substructures due to the limited spatial resolution in the reconstructed SPECT image (partial volume effect, PVE). In order to estimate the extent of underestimation in the present study, the reconstructed spatial resolution was estimated on the basis of a line source measurement using the same acquisition and reconstruction protocol as in the measurements of the striatal phantom and the patients included in the present study (no AC). Spatial resolution was found to be about 8 mm full-width-at-half-maximum (FWHM). Then a high-resolution CT of the striatal phantom was segmented manually (top row; from left to right: transversal, sagittal and coronal slice). Voxel values in the striatum were set to 6.5, voxel values in the background to 1.0 in order to simulate the actual SBR of about 5.5 in the phantom studies (Table 1). Then the segmented CT image was smoothed with a 3-dimensional Gaussian kernel with 8 mm FWHM to simulate the PVE in SPECT (bottom row). ROI analysis of the smoothed image resulted in a striatal SBR of 3.2 which underestimates the actual SBR of 5.5 by about 42%.

The phantom studies demonstrated strong underestimation of the actual SBR between about 48% (Chang AC) and about 57% (no AC) in the reconstructed SPECT images. Simulation showed that this can be explained by partial volume effects to large extent (Fig. 5). The effect of photon attenuation is rather small compared to the partial volume effects.

Assuming that iterative reconstruction modelling attenuation using the CT-based attenuation map provides more accurate correction of photon attenuation in the brain than Chang AC, the latter being based on a strongly simplified model, the results of the present study suggest that Chang AC overestimates attenuation of photons originating from the striatum relative to the attenuation of photons from the reference region. To some extent this might be explained by the use of the narrow-beam LAC µ = 0.148 cm^−1^ for both Chang and CT-based AC, which might be too large, if scatter correction is incomplete. However, Chang AC resulted in larger SBRs than CT-based AC even with LAC as low as µ = 0.10 cm^−1^ (LAC for CT-based AC fixed at µ = 0.148 cm^−1^). Warwick and co-workers also found larger SBRs in dopamine transporter SPECT with Chang AC than with CT-based AC, at least in the patient scans included in their study [35]. Rajeevan et al. evaluated non-uniform AC based on transmission imaging for I-123-β-CIT and concluded that both non-uniform and uniform AC provide a small improvement of semi-quantitative analysis compared to no AC [36].

The effect of the AC method on left/right asymmetry was small (Table 2). This can be explained by the definition of left/right asymmetry as the difference of SBRs within a ROI and its mirror ROI. Effects of AC cancel, because attenuation characteristics are essentially the same in both hemispheres. The effect on the caudate-putamen ratio was also small, which is explained by similar arguments.

Concerning the diagnostic value of the considered semi-quantitative parameters, the putamen SBR provided the best differentiation between normal and reduced DAT availability (with the written report in the patient’s file as gold standard) [37]. The effect of AC was again negligible.

The lack of an impact of AC on the diagnostic power of I-123-ioflupane SPECT was confirmed by the results of the visual interpretation of the SPECT images. Neither the scores of the 6 individual raters nor the consensus scores obtained at each centre showed an effect of AC on their diagnostic value. This finding is in agreement with the results of Bienkiewicz and colleagues who compared Chang AC (with filtered backprojection) and CT-based AC (with iterative OS-EM reconstruction) in I-123-ioflupane SPECT with respect to visual evaluation by a single reader and found no sigificant effect [38].

Agreement of the retrospective binary consensus score with the classification of DAT availability in the written report ranged between 79.6% and 83.3%, which appears rather low. The most frequent discrepancy was that a scan which had been classified as normal in the written report was scored as reduced in the retrospective visual analysis, indicating somewhat more sensitive reading in the restrospective analysis. This might be explained, at least in part, by the fact that only anonymized SPECT images were provided for the restrospective visual reading (Fig. 2), whereas clinical data and semi-quantitative analysis of the I-123-ioflupane uptake had been available in addition to the SPECT images for the classification in the written report in the patient’s file. Furthermore, retrospective visual reading was based on a standardized documentation page presenting exactly the same slices in each individual patient with a fixed, predefined colortable (Fig. 2), whereas there is variation of slice orientation and colortable in visual analysis of SPECT images in clinical routine.

In addition to accuracy, stability is an important attribute of any diagnostic procedure in clinical routine patient care. In case of radionuclide imaging, variability in the interpretation of the images between different readers is most likely the limiting factor rather than variability of tracer uptake between different scans of the same patient [39]. The present study demonstrated very good to excellent inter-rater agreement of visual reading, particularly for the binary differentiation between normal and reduced DAT availability, which is more relevant clinically than grading the reduction of DAT availability using the 5-score based on Benamer et al. [31]. Inter-rater agreement in this study was comparable to results reported by Kahraman and colleagues [40]. The effect of AC on inter-rater agreement was negligible (Table 3).

AC also had no impact on the certainty of visual differentiation between normal and reduced DAT availability. Only the status of DAT availability had an effect: the certainty was significantly higher in patients with reduced DAT availability than in patients with normal DAT availability, as direct consequence of the instruction given to the raters to interpret the SPECT images conservatively, as in clinical routine patient care where it is important to keep the rate of false positive results low.

**Figure 6:**
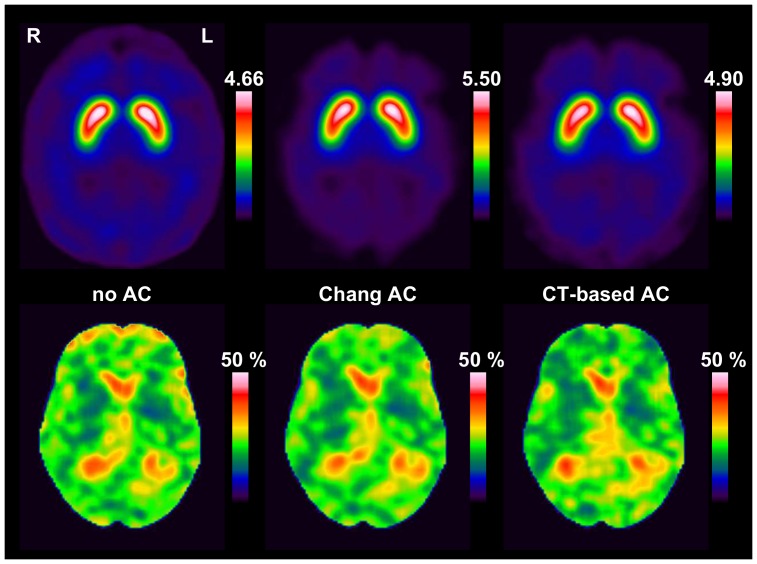
Image quality. Slab of 12 mm thickness of the scaled, stereotactically normalized I-123-ioflupane SPECT averaged over all patients with normal DAT availability (**top**). Slab displaying the coefficient of variation (%) of the DVR over all patients with normal DAT availability (**bottom**).

Finally, the method for AC, i.e. CT-based versus Chang, also had no significant effect on image quality. Image quality appeared slightly worse without AC, most likely because of apparently increased tracer uptake outside the brain which somewhat complicates the identification of the brain (Fig. 6).

The following limitations of the present study should be noted. First, the diagnosis in the original report was used as gold standard for the evaluation of diagnostic accuracy, because a final clinical diagnosis based on follow-up was not available. However, we do not consider this as a major limitation, since the gold standard mainly affects the absolute value of the diagnostic accuracy. Relative diagnostic accuracy is much less affected. The statement that the impact of attenuation correction (with versus without) and the impact of the AC method (CT-based versus Chang) on diagnostic accuracy is negligible, therefore, is not affected. In addition, semi-quantitative analyses (Bland-Altman plots) as well as percent agreement, certainty and image quality according to the visual reading are also not affected by the gold standard.

Second, Chang attenuation correction is a post reconstruction method, i.e. the correction is performed by voxelwise multiplication of the SPECT image reconstructed without attenuation correction with a correction image calculated for a constant attenuation coefficient within the head contour according to Chang [18]. Now, the simplified µ map consisting in a constant value within the head contour might also be used for modelling attenuation within the iterative reconsruction instead of the attenuation map derived from the low-dose CT. It would be interesting to check whether this approach further reduces the difference of SBRs between CT-based AC and AC based on a uniform µ map. However, whereas there might be an effect on absolute values of SBRs, we do not expect an effect on diagnostic value of I-123-ioflupane SPECT, because there is already no difference between CT-based AC and post reconstruction Chang AC with respect to diagnostic value.

## Conclusion

CT-based attenuation correction was compared with Chang attenuation correction with respect to its impact on the diagnostic accuracy of visual and semi-quantitative analysis as well as on inter-rater variability of visual image interpretation in I-123-ioflupane SPECT. These measures are more relevant in clinical routine patient care than absolute quantitative accuracy, as the objective is the discrimination between healthy and diseased state rather than accurate measurement of the density of dopamine transporters (for example in fmol/ml).

The impact of CT-based attenuation correction on the visual interpretation and the diagnostic value of semi-quantitative analysis of I-123-ioflupane SPECT was found to be negligible. Therefore, CT-based attenuation correction cannot be recommended for routine use, not least because of the additional radiation exposure caused by the low-dose CT.
